# IFA-ICP: A Low-Complexity and Image Feature-Assisted Iterative Closest Point (ICP) Scheme for Odometry Estimation in SLAM, and Its FPGA-Based Hardware Accelerator Design

**DOI:** 10.3390/s26082326

**Published:** 2026-04-09

**Authors:** Jia-En Li, Yin-Tsung Hwang

**Affiliations:** 1 Graduate Program in Semiconductor and Green Technology, Academy of Circular Economy, National Chung Hsing University, Nantou 540216, Taiwan; a1085111@mail.nuk.edu.tw; 2 Department of Electrical Engineering, National Chung Hsing University, Taichung 402202, Taiwan

**Keywords:** simultaneous localization and mapping, odometry estimation, image features, Iterative Closest Point algorithm, FPGA, hardware acceleration

## Abstract

Odometry estimation, which calculates the trajectory of a moving object across timeframes, is a critical and time-consuming function in SLAM (Simultaneous Localization and Mapping) systems. Although LiDAR-based sensing is most popular for outdoor and long-range applications because of its ranging accuracy, the sparsity of laser point cloud poses a significant challenge to feature extraction and matching in odometry estimation. In this paper, we investigate odometry estimation from two aspects, i.e., algorithm optimization, and system design/implementation. In algorithm optimization, we present an image feature-assisted odometry estimation scheme that leverages the richness of image information captured by a companion camera to enhance the accuracy of laser point cloud matching. This also serves as a screening mechanism to reduce the matching size and lower the computing complexity for a higher estimation rate. In addition, various schemes, such as adaptive threshold in image feature point selection, principal component analysis (PCA)-based plane fitting for laser point interpolation, and Gauss–Newton optimization for calculating the transform matrix, are also employed to improve the accuracy of odometry estimation. The performance of improved odometry estimation is verified using an existing FLOAM (Fast Lidar Odometry and Mapping) framework. The KITTI dataset for autonomous vehicles with ground truth was used as the test bench. Simulation results indicate that the translation error and rotation error can be reduced by 16.6% and 1.3%, respectively. Computing complexity, measured as the software execution time, also reduced by 63%. In system implementation, a hardware/software (HW/SW) co-design strategy was adopted, where complexity profiling was first conducted to determine the task partitioning and time-consuming tasks are offloaded to a hardware accelerator. This facilitates real-time execution on a resource-constrained embedded platform consisting of a microprocessor module (Raspberry Pi) and an attached FPGA board (Pynq Z2). Efficient hardware designs for customized DSP functions (adaptive threshold and PCA) were developed in an FPGA capable of completing one data frame in 20ms. The final system implementation met the target throughput of 10 estimations per second, and can be scaled up further.

## 1. Introduction

SLAM [1] (Simultaneous Localization and Mapping) is fundamental to applications such as mobile robotics [2,3] and autonomous driving [4]. Its objective is to accomplish localization and mapping when robots/vehicles navigate. Localization enables a vehicle to determine its own position, while mapping involves incrementally building a model of the entire environment by combining sensory data from each scan into a blank map. A typical SLAM architecture can be divided into two sections, i.e., front-end and back-end. The front-end is for real-time state estimation. It processes raw data from sensors like cameras or LiDAR to extract distinctive features. By tracking these features across sequential scans, it computes an estimate of the vehicle’s motion, a process called odometry estimation [5]. This approach is computationally intensive and may suffer from drift, where errors accumulate over time. To remedy this, the back-end focuses on global optimization and consistency. It aggregates the entire history of estimations from the front-end to refine the overall trajectory and map. Its most critical function is Loop Closure Detection [6], which identifies when the vehicle revisits a known location. This optimized trajectory is then used to generate a precise and globally consistent map. The research emphasis of this paper is on the front-end odometry, where hardware acceleration is possible and a balance between computing efficiency and estimation accuracy is sought after.

### 1.1. Investigated Topics and Contributions

Although LiDAR-based sensing is most popular for outdoor and long-range applications because of its ranging accuracy, the sparsity of laser point cloud poses a significant challenge to feature extraction and matching in odometry estimation. In this paper, we present an image feature-assisted odometry estimation scheme that leverages the rich image texture information captured by a companion camera to impose stronger, more diverse criteria for feature selection, enabling the extraction of a more compact and reliable feature set from the outset. This also serves as a screening mechanism to reduce the matching size and lower the computing complexity in laser point cloud matching for a higher estimation rate. The ultimate goal is to achieve the best trade-off between estimation accuracy and computing complexity. Various optimization schemes, such as adaptive threshold in image feature points selection, PCA (principal component analysis)-based [7] plane fitting for laser point interpolation, and Gauss–Newton optimization [8] for calculating the transform matrix, are employed to improve the accuracy of odometry estimation. We also utilize the Ceres Solver [9] to simplify pose estimation. The performance of improved odometry estimation is verified using a complete SLAM framework. The KITTI vision benchmark suite [10] for autonomous vehicles with ground truth was used as the test bench.

After algorithm development, efficient system implementation issue was investigated. A HW/SW (hardware/software) co-design strategy was adopted, which implements massive but regular computing in hardware for processing acceleration and accomplishes the rest by using software for system flexibility. Complexity profiling was first conducted to determine the task partitioning between hardware and software sections. The system was implemented on a resource-constrained embedded platform to substantiate the claims of low complexity and improved accuracy. The target computing platform consisted of a microprocessor-based Raspberry Pi module (for host and software execution) and an FPGA board (for hardware acceleration). Efficient hardware accelerator designs were developed by using the algorithm mapping scheme. Finally, the entire system was set up, and the throughput rate enhancement was measured.

The contributions of this study include the following: (1) In the algorithm aspect, we developed an image feature-assisted scheme to significantly reduce the computing complexity while maintaining the estimation accuracy. (2) In the hardware acceleration aspect, we successfully converted computing-intensive DSP modules (e.g., adaptive threshold and principal component analysis) into efficient hardwired systolic array design. (3) In the system implementation aspect, we realized a complete odometry estimation system on a cost-effective platform following the HW/SW codesign strategy.

### 1.2. Literature Review

Various odometry estimation and mapping algorithms have been reported. To align with our system setting, we will focus on those LiDAR-centric approaches. For instance, LOAM (Lidar Odometry and Mapping) [11] improves stability in pose estimation by segmenting point clouds into edge and planar features. It then performs a two-stage matching and pose estimation process, first against the previous frame and then against the global map. FLOAM (Fast Lidar Odometry and Mapping) [12] extends the work of LOAM’s odometry estimation by matching points only against the global map to expedite the process without a significant compromise in accuracy. In a different approach, KISS (Keep It Small and Simple)–ICP (Iterative Closest Point) [13] introduces the concept of adaptive thresholding to mitigate the efforts of manual parameter tuning. Recently, multi-sensor fusion has emerged as a viable option for achieving high-precision localization and mapping. For example, the LIMO (Lidar-Monocular Visual Odometry) [14] system fuses LiDAR with a monocular camera and leverages LiDAR’s precise ranging capabilities to aid in depth estimation for image pixels. The image feature-assisted selection mechanism adopted in this paper is inspired by the fusion concepts of LIMO and the adaptive scheme of KISS-ICP. The Traj-LIO (LiDAR-Inertial Odometry) [15] algorithm integrates an IMU (Inertial Measurement Unit) to augment its LiDAR system and introduces the concept of a continuous-time trajectory to effectively mitigate motion distortion in LiDAR scans. Although rotational and translational components in its Gaussian process inference are decoupled in Traj-LIO, its overall computational load remains considerable.

From the preceding analysis, a clear trade-off spectrum emerges: LOAM-based methods strive for ultimate precision at a high computational cost; KISS-ICP offers adaptability within a point-to-point matching paradigm, while LIMO and Traj-LIO achieve superior robustness through complex multi-sensor architectures. Therefore, in this research, FLOAM is adopted as a strategic starting point because it retains the robust feature of LOAM but streamlines it into a single-stage odometry pipeline.

The rest of the paper is organized as follows: In Section 2, the proposed odometry estimation framework is described, followed by the associated simulation results and performance analyses in Section 3. In Section 4, the system implementation, based on the concept of HW/SW codesign, is described. In addition, hardwired logic designs to accelerate time-consuming modules are elaborated.

## 2. Proposed Odometry Estimation Algorithm of IFA-ICP

In this section, we will elaborate the proposed image feature-assisted–Iterative Closest Point (IFA-ICP)-based odometry estimation scheme.

### 2.1. Image Feature-Assisted Point Cloud Screening and Feature Extraction

**Figure 1:**
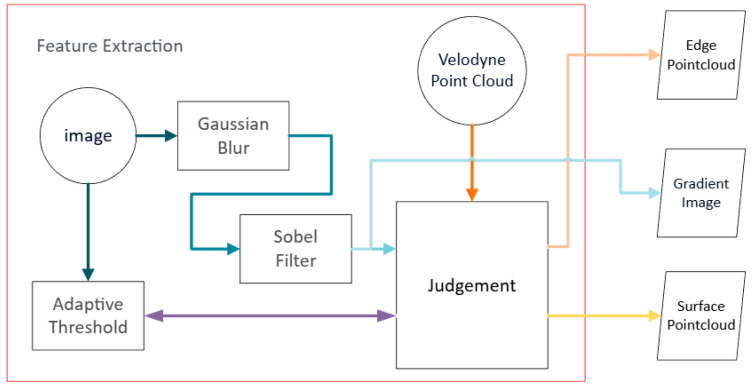
Flowchart of image feature extraction module.

The proposed image feature-assisted extraction process starts with the pre-processing of real-time image frames. The goal of this process is to extract prominent laser points with either edge or plane features in the 3D spatial domain by way of leveraging texture information in the 2D image domain. Figure 1 depicts a flowchart of the image feature-assisted feature extraction, and the corresponding pseudo code is listed in Algorithm 1. A Gaussian Blur [16] process is first applied to lower the noise impact, followed by the application of a Sobel Filter [17] to generate a gradient map. To facilitate the fusion of image and LiDAR data, the 3D LiDAR point cloud is projected onto the 2D image plane. This projection is achieved by first transforming the point cloud from the LiDAR to the camera coordinate system using a pre-calibrated Extrinsic Matrix and then mapping the 3D points to their 2D pixel coordinates via the camera’s Intrinsic Matrix. Let $p={\left[x,y,z\right]}^{T}$ denote a 3D point in the LiDAR coordinate system. Its corresponding 2D pixel site in the camera coordinates can be calculated as follows:

(1) $$\left[\begin{array}{c} u^{\prime }\\ v^{\prime }\\ w^{\prime } \end{array}\right]=K\cdot E\cdot \left[\begin{array}{c} x\\ y\\ \begin{array}{c} z\\ 1 \end{array} \end{array}\right]$$

Here, $E\in R^{3\times 4}$ is the Extrinsic Matrix that transforms points from LiDAR to camera coordinates and $K\in R^{3\times 3}$ is the camera’s Intrinsic Matrix. This projection function can be concisely represented as $p_{x}=\pi (K,E,p)$, where $p_{x}={\left[\frac{u^{\prime }}{w^{\prime }},\frac{v^{\prime }}{w^{\prime }}\right]}^{T}$. This function corresponds to row 7 of Algorithm 1. Note that this projection is to find the correspondence of local coordinate systems between the camera and LiDAR. There is a global coordinate system for the constructed map consisting of past LiDAR points.

Each LiDAR point of the current frame, after being projected to the image domain, is classified as either an edge or a planar feature by using the surrounding image texture information. Traditional image-based methods, such as Canny Filter [18], rely on a single, global threshold and thus lack adaptability to dynamic scenes with varying illumination and textures. We overcome this limitation by employing a simplified adaptive threshold [19] algorithm. For each projected point, a 3 × 3 neighborhood is sampled from the original image. Pixel values of each of the three columns are sorted to find their respective maximum, median, and minimum. The adaptive threshold for the central pixel is then computed as the arithmetic mean of three values: the minimum of the three maximums, the median of the three medians, and the maximum of the three minimums. For a projected point at pixel coordinate $p_{x}$, let its 3×3 neighborhood in the original image $I_{curr}$ be denoted by a matrix $N(p_{x})$. Let the three columns of this matrix be ***c*_1_, *c*_2_,*c*_3_**. The adaptive threshold $t_{edge}(p_{x})$ is calculated as

(2) $$t_{edge}(p_{x})=\frac{1}{3}(\underset{j\in \{1,2,3\}}{\min}\left\{\max\left(c_{j}\right)\right\}+\underset{j\in \{1,2,3\}}{\mathrm{median}}\left\{\mathrm{median}\left(c_{j}\right)\right\}+\underset{j\in \{1,2,3\}}{\max}\left\{\min\left(c_{j}\right)\right\})$$

This function corresponds to row 9 of Algorithm 1.

During classification, a point’s gradient value from the Sobel map is compared against this locally computed adaptive threshold. The corresponding LiDAR point is labeled as an edge feature if its gradient exceeds the threshold, and as a planar feature if its gradient is substantially lower (e.g., below 25% of the threshold). Let $g_{val}\left(p_{x}\right)$ be the gradient value at pixel $p_{x}$ from the Sobel gradient map $I_{grad}$. The classification of the corresponding 3D point p into the edge feature set $F_{edge\_curr}$ or the surface feature set $F_{surf\_curr}$ is determined by the following rule:

(3) $$p\in \{\begin{array}{c} F_{edge\_curr}\;\;\;\;if\;g_{val}\left(p_{x}\right)>t_{edge}(p_{x})\;\\ F_{surf\_curr}\;\;\;\;if\;g_{val}\left(p_{x}\right)<\;\alpha \cdot t_{edge}(p_{x}) \end{array}$$

where α is a coefficient set to 0.25 in our implementation. Points whose gradient values fall in between are discarded. This function corresponds from row 12–17 of Algorithm 1. The benefit of this approach is that the classification can be performed in a 2D image domain with rich texture information rather than in a 3D spatial domain relying on structural information only. The latter poses a more challenging task.
**Algorithm 1.** Algorithm of image feature-assisted feature extraction.**Feature Extraction Scheme of IFA-ICP**1.**procedure** FeatureExtraction(P_curr, I_curr)2.     I_blur ← GaussianBlur(I_curr)       /* Gaussian Blur operations */3.     I_grad ← SobelFilter(I_blur)          /* Sobel filtering */4.     F_edge_curr ← Ø                             /* initialize set of edge feature points */5.     F_surf_curr ← Ø                            /* initialize set of surf. feature points */6.     **for each** point p in P_curr **do**7.         px ← ProjectToImage(p, K, E)     /* projecting laser point to image */8.         **if** px is within I_curr bounds **then**9.             t_edge ← CalculateAdaptiveThreshold(I_curr, px)10.             t_surf ← t_edge / 411.             g_val ← GetValueAt(I_grad, px)  /* gradient value of projected point*/12.             **if** g_val > t_edge **then**13.                  Add p to F_edge_curr14.            **else if** g_val < t_surf **then**15.                  Add p to F_surf_curr16.
                  **end if**
17.             **end if**
18.          end for19.     **return** F_edge_curr, F_surf_curr I_grad  /* return two feature point sets */20.     end procedure

### 2.2. Interpolated Feature Point Matching for Odometry Estimation

**Figure 2:**
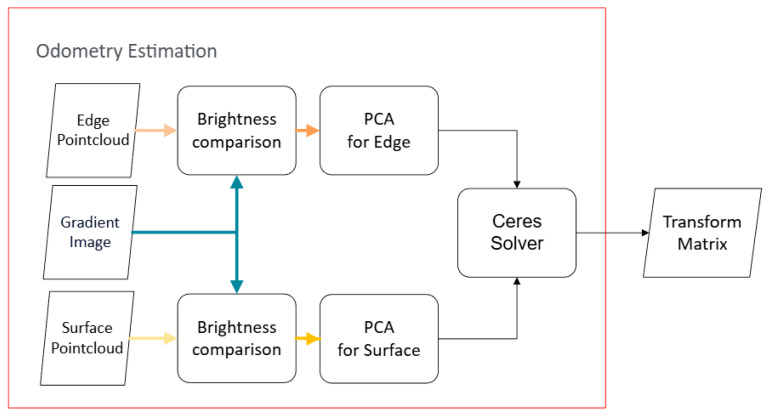
Flowchart of the IFA-ICP based odometry estimation module.

In the odometry estimation stage, the core task is to solve the pose transformation between two successive scanning frames by matching the feature points of the current frame against an existing (or constructed) global map. Figure 2 shows a flowchart of the IFA-ICP based odometry estimation scheme. The inputs edge point cloud, surface point cloud, and gradient image are obtained from the image feature extraction module, as shown in Figure 1. Edge and planar feature points are processed separately. The pose transformation matrix is usually calculated by performing an ICP (Iterative Closest Point) [20] scheme. However, as previously mentioned, the scanning pattern of mechanical LiDAR results in point clouds is inherently sparse in vertical dimensions. The implication is that there are good chances of no matched LiDAR point pair between them. To tackle this issue, the LOAM algorithm introduces an interpolation strategy during its ICP process and verifies the correctness of matches by examining the geometric structure of the matched point set (e.g., whether they form a line or a plane). Building upon this geometric feature matching philosophy, our research introduces a key refinement: when matching the edge and planar points extracted in the previous stage, the system first finds the five nearest neighbors in the local map for each feature point. While LOAM and FLOAM would proceed directly to PCA (Principal Component Analysis) on these five neighbors to verify their geometric structure, our method introduces a pre-filtering step based on image grayscale values before the PCA. This is designed to improve matching efficiency by rejecting false matches early on. However, although we can easily obtain the grayscale value for a feature point in the current frame, we cannot directly acquire the grayscale for a map point (an existing point in the constructed map) in the current image because the current camera pose is not yet known. Our solution is to project the map points onto the image from the previous timestep. Specifically, we calculate the centroid of the five neighboring map points and project this centroid onto the previous image plane as an approximation. The grayscale of this projected map point is then compared to that of the current feature point. If the discrepancy is significant, the match is most likely incorrect and is discarded immediately. Conversely, if the grayscale values are similar, the pair is considered a reliable candidate match and is passed to the subsequent geometric verification stage. The rationale behind this is based on the photo-consistency of image sequences if the framerate is sufficiently high. The relative motion between frames is small enough that the grayscale value of a physical landmark remains stable. Furthermore, the centroid approach acts as a spatial low-pass filter, mitigating the impact of LiDAR measurement noise and ensuring that the pre-filtering step is robust against minor projection misalignments. The goal of this method is to streamline computationally expensive geometric verification with fewer but more accurate points, not to output the ultimate pose solution.

This process is further elaborated as follows: For a current feature point $f_{c}$ with brightness ${b(f}_{c})$, let its 5 nearest neighbors in the local map be the set $N=\{m_{1},\ldots ,m_{5}\}$. The centroid of these neighbors is $\bar{m}=\frac{1}{5}\sum_{i=1}^{5}m_{i}$. The match is considered valid and proceeds to geometric verification only if the brightness difference is below a threshold $\tau_{b}$:

(4) $$\left|{b(f}_{c}\right)-b_{prev}(\pi (K,E_{prev},\bar{m}))|<\tau_{b},$$

where $b_{prev}(\cdot )$ retrieves the brightness from the previous image frame $I_{prev}$, and π(·) is the projection function defined earlier, using the previous frame’s pose $E_{prev}$.

Algorithm 2 shows the pseudo code of the proposed IFA-ICP-based odometry estimation algorithm. Edge and planar feature points are first constructed into separate KD (K-dimensional) trees [21], respectively. For a candidate edge point, PCA is performed on its five neighbors. If their eigenvalue distribution indicates a distinct linear structure, the match is considered successful, and the point-to-line distance is calculated as the residual. For a candidate planar point, PCA is used to find the best-fit plane. If all five neighbors lie within a certain distance of this plane, the match is considered successful, and the point-to-plane distance is calculated as the residual. The definition of the point-to-line residual is shown in Equation (5):

(5) $${residual}_{edge}=\frac{\left|(f_{e}-m_{a})\times (f_{e}-m_{b})\right|}{\left|m_{b}-m_{a}\right|}$$

Here, $f_{e}$ represents the edge point in the current scanning. $m_{a}$ is the centroid plus the eigenvector corresponding to the largest eigenvalue in PCA, and $m_{b}$ is the centroid minus the eigenvector corresponding to the largest eigenvalue. This function corresponds to row 10 of Algorithm 2.

The point-to-plane residual is shown in Equation (6):

(6) $${residual}_{surf}=|(f_{s}-m_{p}{)}^{T}\cdot n|$$

Here, $f_{s}$ is the planar point in the current scanning, $m_{p}$ is the centroid used in the pre-filtering stage, and n is the eigenvector corresponding to the smallest eigenvalue in PCA. This function corresponds to row 19 of Algorithm 2.

The odometry estimation is formulated as a non-linear least-squares optimization problem. The goal is to find a rigid-body transformation T=[R|t] (composed of rotation R and translation ***t***) that minimizes the sum of squared residuals from all valid edge and surface feature matches:

(7) $$\underset{T}{\mathrm{argmin}}\left(\sum_{f_{e}\in F_{edge}^{\prime }}d_{e}{\left(T\cdot f_{e},N_{e}\right)}^{2}+\sum_{f_{s}\in F_{surf}^{\prime }}d_{s}{\left(T\cdot f_{s},N_{s}\right)}^{2}\right)$$

where $F_{edge}^{\prime }$ and $F_{surf}^{\prime }$ are the set of feature points passing both the brightness pre-filtering and geometric verification stages. The function d(·) calculates the point-to-line or point-to-plane distance for the transformed feature point T·f. In our algorithm, this optimization problem is solved using the Ceres Solver. This function corresponds to row 23 of Algorithm 2.
**Algorithm 2.** Algorithm of image feature-assisted odometry estimation.**IFA-ICP based Odometry Estimation Algorithm**1.**procedure** OdometryEstimation(F_edge_curr, F_surf_curr, M_edge_prev, M_surf_prev, I_grad)    /* KD-Tree construction [21] */2.    KDTree_edge ← BuildKDTree(M_edge_prev) 3.    KDTree_surf ← BuildKDTree(M_surf_prev)4.    Residuals ← Ø    /* module for operations on edge feature points */5.    **for each** feature f_e in F_edge_curr **do**6.       N_e ← KDTree_edge.FindNearestNeighbors(f_e, 5) // find 5 nearest points 7.       **if** BrightnessCheck(f_e, N_e, I_grad) is true **then**  /* check if 5 points of                                                                                                    similar gray values */8.            isLine, lineParams ← PCA_Validate_Line(N_e) /* PCA to validate if                                                                                                    5 points in a line */9.            **if** isLine is true **then**10.                Add PointToLineResidual(f_e, lineParams) to Residuals11.
            **end if**
12.
        **end if**
13.    end for
    /* counterpart module for operations on surface feature points */ 14.    **for each** feature f_s in F_surf_curr **do**15.        N_s ← KDTree_surf.FindNearestNeighbors(f_s, 5)16.        **if** BrightnessCheck(f_s, N_s, I_grad) is true **then**17.                        isPlane, planeParams ← PCA_Validate_Plane(N_s)18.            **if** isPlane is true **then**19.                        Add PointToPlaneResidual(f_s, planeParams) to Residuals20.                   end if21.            end if22.        end for23.        ΔT ← SolveLeastSquares(Residuals)24.        **return** ΔT25.end procedure

## 3. Performance Analyses and Simulation Results

### 3.1. Algorithm Simulation Setting

The performance of the proposed algorithm was empirically evaluated on an experimental platform consisting of a high-performance laptop (spec. in Table 1). We selected the KITTI dataset, which is a cornerstone benchmark for autonomous driving research, as our primary test pattern. This dataset consists of real-world driving scenarios and provides precise ground truth trajectories for certain sequences so that quantitative validation of our method’s accuracy can be conducted.

Our system is built upon the ROS (Robot Operating System) [23], which is an open-source meta-operating system. We leverage its distributed architecture to modularize the complex SLAM pipeline into independent nodes, which can communicate with each other via an asynchronous publish-subscribe topic system. To meet the stringent real-time processing requirements inherent to SLAM, all core algorithmic components were implemented in C++ for maximum performance and stability. In our pre-processing stage, we also address the issue of motion distortion in the LiDAR scans. The same real-time de-skewing technique as FLOAM was employed to correct for the point cloud deformation caused by ego-motion. Specifically, to ensure a fair comparison, all evaluated algorithms were constrained to only process the frontal point cloud data, similar to a view scope spanned by a forward-facing camera.

### 3.2. Simulation Results and Accuracy Comparison

**Figure 3:**
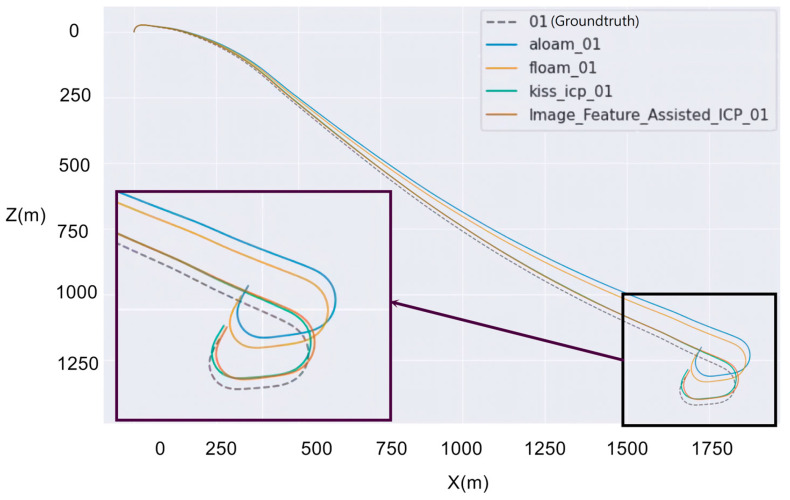
Endpoint path of each algorithm in sequence 01.

This section presents a quantitative comparison of the proposed algorithm with other state-of-the-art methods, focusing on the evaluation of estimation accuracy. The trajectory errors presented herein were computed using the Python script provided by the authors of [24]. In addition to the proposed one, three other schemes, i.e., A-LOAM (Advanced implementation of LOAM) [16], FLOAM [3], and KISS-ICP [4], are included. Note that A-LOAM [25] is an improved version of LOAM [11]; it keeps the original algorithm, but utilizes optimized libraries like Eigen and Ceres Solver. In this study, LiDAR information is the backbone of localization, and image information is only the auxiliary measure. Visual or visual–inertial SLAM systems such as ORB-SLAM [26] adopt a different approach to using image information: they do not rely on LiDAR for ranging, but require image-based depth estimation, which is more effective in short-distance ranging where the disparity of objects from either binocular cameras or across different timeframes is more prominent. Because of the fundamental difference in sensing modality, they are not included in this comparison. Table 2 lists the simulations when evaluated on sequence 01. Our method yields a substantial enhancement in translation over competing methods. The endpoint resulting trajectory for this sequence is visualized in Figure 3, which was plotted using the evaluation tool from [27].

**Figure 4:**
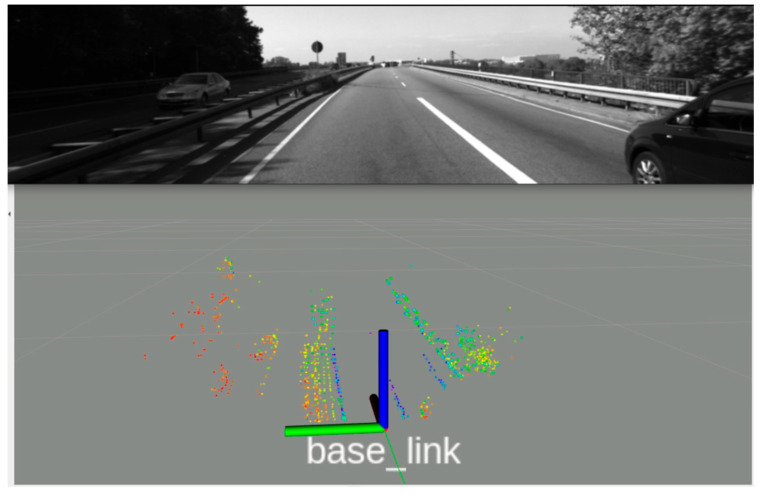
Extracted feature points by the proposed scheme in a scene of sequence 01.

To ascertain the underlying cause of this discrepancy, we examined the specific trajectory segments where competing algorithms exhibited significant drift. It became apparent that a critical ~300 m section of road bordered by dense vegetation (Figure 4) was the main cause. In traditional LiDAR-only odometry, laser returns from foliage are frequently misclassified as stable edge features based on their geometry alone. However, since vegetation is non-rigid and its appearance lacks geometric consistency across scans, these unreliable pseudo-features create ambiguity in the matching process, leading to the accumulation of odometry errors. By integrating visual data into the feature extraction pipeline, our scheme can downweight or filters out these unstable elements prior to matching. The efficacy of this approach is visually evident in Figure 4 (bottom), where the point cloud processed by our scheme was largely purged of noise from the surrounding bushes and the number of false features was drastically reduced.

Table 3 lists the detailed accuracy comparison results of 11 sequences in the KITTI dataset. Since the proposed scheme was developed on the basis of FLOAM, it was used as a reference to evaluate the effectiveness of the modifications introduced in the proposed scheme. Entries marked in red indicate that the proposed scheme outperforms the original scheme. On average, the translation error reduced by 16.6% and the rotation error reduced by 1.3%.

### 3.3. Execution Time Comparison

Regarding the computing complexity comparison, execution time was used as the index, and the results are shown in Table 4. Note that the experiments were conducted on a PC (x86) platform, not on the target implementation platform. The execution time of our scheme was normalized to 1. Our proposed algorithm achieved a speedup of an order of magnitude relative to the LOAM family. Even against FLOAM, our method can reduce computation time by roughly 70%. While KISS-ICP stands out for its excellent balance of accuracy and speed, our system surpasses it in efficiency as well. Our method reduces the computational time by nearly 50% compared to KISS-ICP. Based on the above comparison results, our approach realizes a superior level of computational efficiency without compromising its high accuracy.

**Figure 5:**
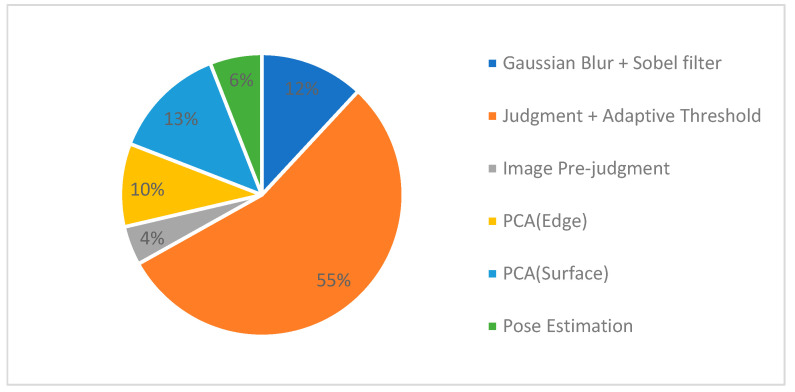
Computing complexity breakdown of the proposed scheme on the PC(x86) platform.

This performance gain is primarily attributed to our novel front-end design. By strategically reducing the number of points handled in the initial stage, our method drastically lowers the computational load on downstream tasks like feature matching and pose optimization, thereby boosting the entire system’s throughput. Figure 5 illustrates the computing complexity breakdown of the proposed scheme. The judgment and adaptive thresholding tasks in the feature point extraction module account for more than 50% of the total computing complexity. Along with Gaussian blur and Sobel filtering, the entire feature extraction module consumes 67% of computation. This, however, alleviates the computing complexities of the downstream modules significantly.

Compared to the LiDAR-only FLOAM, the proposed image-assisted screening drastically prunes unreliable candidates. While traditional geometric methods often misclassify non-rigid objects (such as dense vegetation) as stable features, our targeted filtering method leverages visual texture to eliminate these spurious points early in the pipeline. To quantify the effectiveness of this screening process, an extensive evaluation was conducted across four KITTI sequences (00, 01, 02, and 06). The average number of LiDAR points for Ceres Solvers in calculating the transformation matrix was used as the evaluation metric. As shown in Table 5, the image-assisted mechanism significantly optimizes the search space for the pose estimation solver. On average, the number of candidate edge points reduced by 78.85%, while the surface feature set was pruned by 72.72% This consistent reduction across diverse environments demonstrates the robustness of our screening mechanism in minimizing computational complexity without compromising estimation accuracy.

## 4. System Implementation Based on Hardware/Software Codesign

After algorithm development, we focused on its system implementation. Real-world applications, such as cost- and power-constrained automotive or robotic platforms, still present challenges: the systems need to operate in such resource-constrained environments and meet real-time requirements. We will next explore a HW/SW co-design approach, which aims to offload the computational bottlenecks to a dedicated hardware accelerator for speedup purpose, while realizing the rest of the system on a software-driven computing platform for flexibility.

### 4.1. System Design Specifications

The goal of this research is to validate that our proposed high-performance SLAM algorithm can be successfully deployed on a low-cost, low-power embedded platform and operate in real-time without imposing an excessive burden on the overall system. To establish a quantitative benchmark, we set a real-time processing target of 10 frames per second (FPS), which is also the evaluation benchmark used by the KITTI dataset. Although our algorithm can exceed 60 FPS on an Intel Core i7 platform, the true challenge lies in meeting the stringent 10 FPS timing requirement on a severely resource-constrained platform.

The HW/SW co-design verification platform we used features a Raspberry Pi 4 [28] as the main host processor and a PYNQ-Z2 (FPGA) [29] as a co-processor, as listed in Table 6. Both are widely adopted platforms in academia and industry. This combination embodies the heterogeneous computing architectures common in modern embedded systems. Since critical functions are implemented in hardware for acceleration, this can greatly alleviate the computing efforts on a programmable processor. No high-end CPU or GPU is required, and a low-cost microprocessor-based module suffices for this purpose. On the other hand, the limited computational power of the RPi 4 intentionally highlights the performance bottlenecks of a software-only solution, thereby proving by contrast the necessity and effectiveness of hardware acceleration. As for the FPGA platform, while it is possible to integrate everything into a high-end FPGA device, the cost and power consumption might not be appealing from the aspect of system implementation. The final implementation results also indicate that the capacity of PYNQ-Z2 is sufficient to implement the hardware accelerator.

### 4.2. HW/SW Codesign System Planning and Timing Analysis

To determine the HW/SW task partitioning, the performance of the proposed SLAM algorithm was first benchmarked in a pure software configuration on Raspberry Pi 4, the target software execution unit in the target implementation platform. Sequence 04 of the KITTI dataset was used as the test bench. The computing resource of the ARM processor in Raspberry Pi 4 is much lower than that of the intel x86 CPU. We need the real execution time of each module in performing time budgeting and task partitioning to make sure that the 10 FPS throughput rate (100 ms in latency) can be met. In addition to high computing complexity, a computing task must be regular enough to benefit from the hardware acceleration.

Table 7 shows the profiling results and lists the execution time of each module. The sum-up time was 152.78 ms, which corresponds to the execution time for calculating a pose transformation matrix. This equates to a processing throughput of 6.54 FPS. The last column of the table indicates the time-consuming modules to be expedited by hardware accelerators. Note that, in our final implementation, the “Adaptive Threshold” part is implemented in hardware while the “judgement” remains in software.

The entire system is built around the ROS ecosystem, which provides essential infrastructure, from communication protocols and time synchronization to visualization tools like Rviz. A host platform running a full ROS instance managed high-level scheduling and data flow, while computationally intensive tasks were transmitted via TCP over Ethernet to an FPGA co-processor for acceleration. The results were then returned via TCP.

The performance profile of the software implementation reveals three primary computational bottlenecks: the adaptive threshold calculation, KD-Tree construction, and the Principal Component Analysis (PCA) of geometric features. Among them, KD-Tree construction is an inherently sequential process. While some intra-level parallelism exists, the algorithm fundamentally requires log(N) sequential steps. A fully parallel hardware implementation would be infeasible. Therefore, we focused hardware acceleration on the two other bottlenecks: the adaptive threshold and PCA computations. These modules are not only computationally demanding, but also exhibit a structure far more amenable to efficient hardware parallelization.

### 4.3. HW/SW Communication Protocol and Computing Synergy

**Figure 6:**
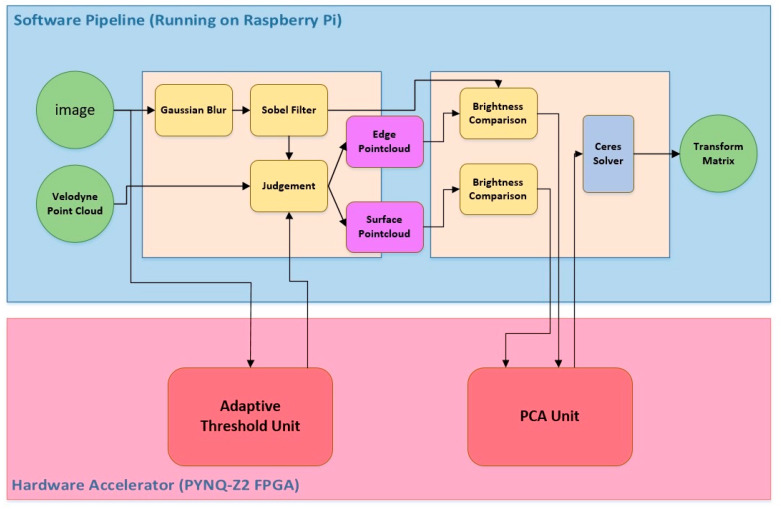
Illustration of SW and HW computing synergy.

Communication architecture is a cornerstone of any HW/SW co-design system, demanding a balance between efficiency and reliability. The acceleration of tasks like the adaptive threshold and PCA involves transaction-based transfers of ~500 KB datasets. Therefore, we selected the TCP protocol for its inherent reliability, guaranteeing error-free data delivery to the hardware. Figure 6 illustrates the interaction between the SW section and HW section. Upon connection between Raspberry Pi (host) and PYNQ (client) modules, the host sends data packets for processing. The PYNQ’s Processing System (PS) is configured to act as a TCP server, receives these packets via its TCP/IP stack, and stages the data in DDR memory. To initiate hardware acceleration, the PS then configures and triggers a DMA controller, which autonomously transfers the prepared data from the DDR to our custom accelerator IP in the Programmable Logic (PL) through a high-throughput AXI-Stream interface. The accelerator IP and its data path are designed to operate at 125MHz, which is the native clock frequency of the PYNQ-Z2’s ethernet subsystem. Following the computation by the hardware IP, the results are returned to the host client, completing the full HW/SW interaction cycle.

### 4.4. Time Budgeting and Task Scheduling

Our performance target was to achieve a real-time throughput of 10 FPS for the SLAM front-end, meaning that one pose transformation must be computed within 100 ms to match standard LiDAR update rates. To assess the feasibility of this target, we first quantified the time consumed by essential software tasks and data transfer, which in turn defined the available time budget for parallel software-hardware execution. The transferred data included a grayscale image (1 byte/pixel) and the LiDAR point cloud, which was converted to integers (int, 12 bits/point) to accommodate fixed-point arithmetic in hardware. The data volume for this transfer test was set to 1000 feature points. This number was chosen based on the results from previous algorithm validation experiments, in which the peak number of edge points extracted in a single frame was less than 1000. For this 1000-point test, each data packet for upload contains five neighboring points, one centroid, and the feature point itself. For download, the data for an edge feature includes two residual points and the feature point’s data, while a planar feature includes the plane equation and the feature point. To simulate the maximum data load, we used the larger data packet size of the edge feature for our test. The data transfer times for adaptive threshold, ICP (edge), and ICP (surface) were 5.173 ms, 5.094 ms, and 7.966 ms, respectively.

**Figure 7:**
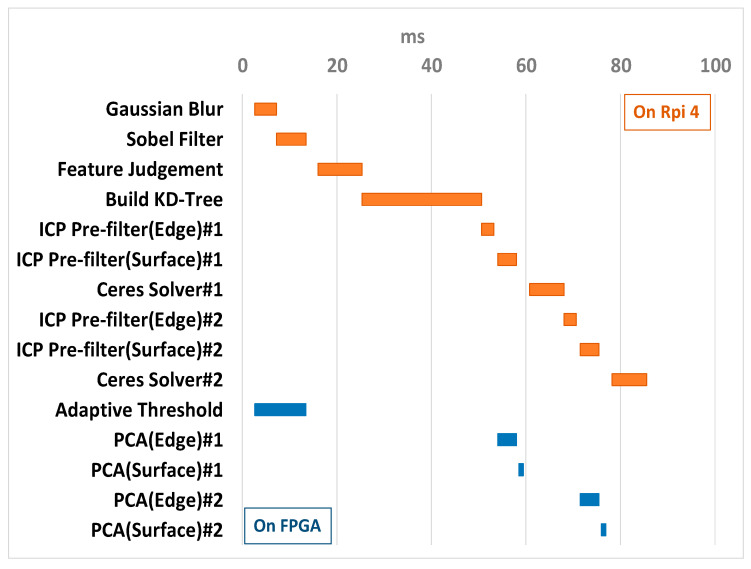
HW/SW codesign system timing Gantt chart.

By combining the runtimes of the software modules and the TCP transfer time, we determined that the available time budget for the hardware acceleration unit and created a Gantt chart, as shown in Figure 7, to plan the execution flow of each module. The top of the chart indicates the software execution schedule performed in Raspberry Pi 4, while the bottom part corresponds to the hardware execution schedule in FPGA (PYNQ-Z2). This chart also reveals the computing parallelism between the hardware and software sections.

### 4.5. Finite Precision Analysis of Fixed-Point Hardware Implementation

We will next elaborate on the hardware accelerator designs. A systematic finite precision analysis is essential before migrating a floating-point version algorithm to a fixed-point hardware (RTL) implementation. This analysis serves two purposes: first, to identify the optimal fractional bit width that balances numerical accuracy against hardware resource utilization; and second, to generate a Golden Pattern for the functional verification of the final RTL design. For iterative algorithms, such as the Jacobi method [30] used in the PCA module, the number of iterations presents a key trade-off between computational cost and precision. Our experiments reveal that, for the Jacobi method, the accuracy gain diminished significantly after the second iteration, indicating that more iterations were not necessary. The impact of quantization errors due to fixed point formats were evaluated using the system’s final translational and rotational errors as the primary metrics. The analysis was tailored to each module. For each computing variable, we first determined its precision of integral part, i.e., the left boundary of the precision window, so that no overflow occurred. The fractional part precision, i.e., the right boundary of the precision window, was next determined to achieve a balance between accuracy and hardware complexity.

**Figure 8:**
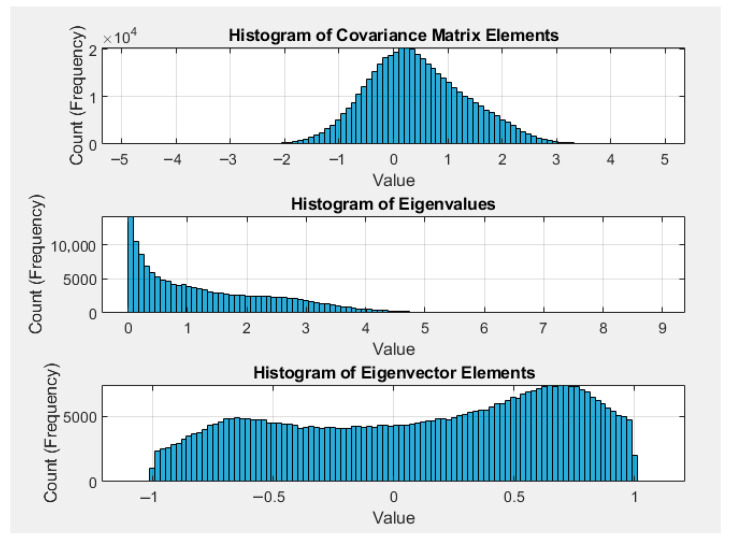
Integer precision analysis based on the histogram of computing variables.

For the integral part analysis, we use the Jacobi module as an example. The input neighbor points were already centralized (mean-subtracted), and the distance of each point from the feature point did not exceed 1 m. Under this condition, 50,000 sets of random data were generated in MATLAB 9.8 R2020a [31] for simulation. As shown in Figure 8, the simulation results indicate that the maximum integer part of the covariance matrix is 4, while the maximum integer value of the eigenvalues is 8. The eigenvectors were normalized to be no greater than 1 due to the nature of Jacobi eigenvalue decomposition. Since the eigenvalues have the largest dynamic range among all values (approaching 9), we need at most 4 bits for the integer part.

**Figure 9:**
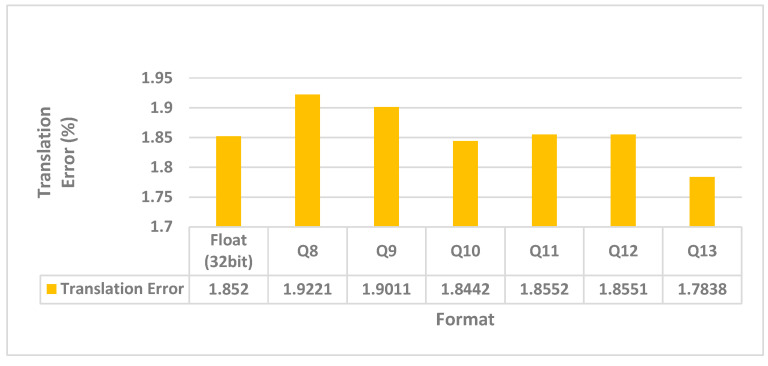
Fixed-point analysis for adaptive threshold module.

For fractional precision analysis, Figure 9 shows the experimental results of the adaptive threshold module. The number of fractional bits vary from 8 to 19, and the corresponding translation errors were calculated and compared with the original (floating point) one. This indicated that the Q10 format [32] (i.e., 10 fractional bits) is the optimal choice. The marginal improvement in accuracy by further increasing the bit width did not justify the substantial increase in hardware resources. Note that the translation error of Q10 (1.8442) is even smaller than that of the floating-point version (1.845). This is not always the case because translation errors vary in different sequences and may not decrease monotonically with the fractional bit width. Based on this conclusion, we will adopt the Q10 format for the subsequent hardware module mapping and RTL design. Note that the simulation results do not simply follow the “more bits, higher accuracy” intuition because of the non-linear accumulation and cancelation of errors.

### 4.6. Hardware Accelerator Designs

**Figure 10:**
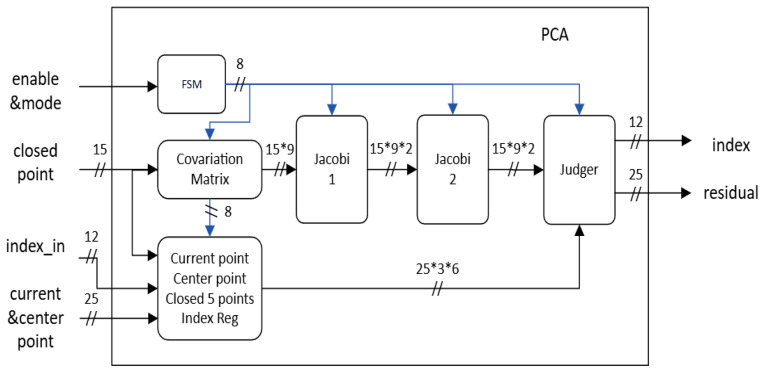
PCA top-level hardware architecture diagram.

There are two types of hardware accelerators: one is adaptive threshold calculation, and the other is PCA. Due to space limitations, we will elaborate the PCA design only. Figure 10 illustrates the top-level architecture of our designed PCA module, which can be divided into two primary sections: a control path and a data path. The core of the control path consists of a Finite State Machine (FSM) and a mode selection signal determining whether the data being processed is for a plane or an edge. As indicated by the blue signal flow in Figure 10, the FSM is responsible for generating a sequence of control signals. To maximize throughput, the data path was implemented as a four-stage pipelined architecture, consisting of covariance matrix calculation, two Jacobi eigenvalue units, and a final classification stage.

**Figure 11:**
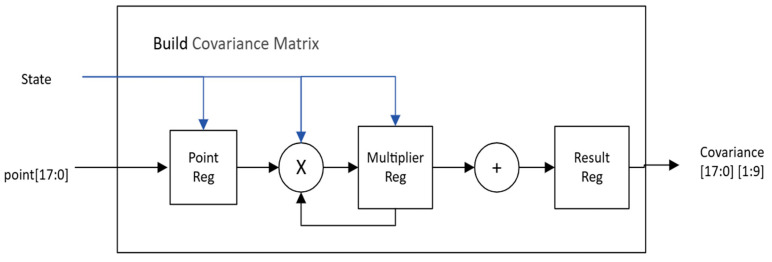
Covariance matrix calculation module.

We will next explain the function and architecture of the sub-circuits. Figure 11 shows the design of covariance matrix calculation, which is essentially an outer product operation requiring multiplications and additions. Since this is not the critical computing of the PCA module, one multiplier and one adder suffice for the real-time requirement. It takes a total of 88 clock cycles to complete the calculations needed for one feature point. For a maximum of 1000 feature points, the computing latency (@125 MHz clock frequency) is 0.704 ms, which is less than the allotted time slot 0.9335ms.

**Figure 12:**
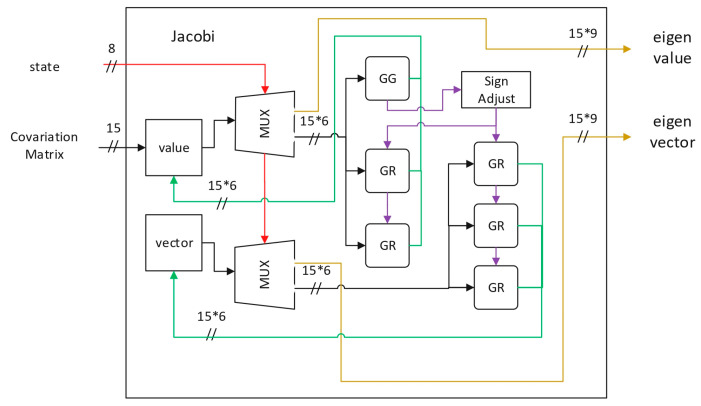
System block diagram of the Jacobi module design.

**Figure 13:**
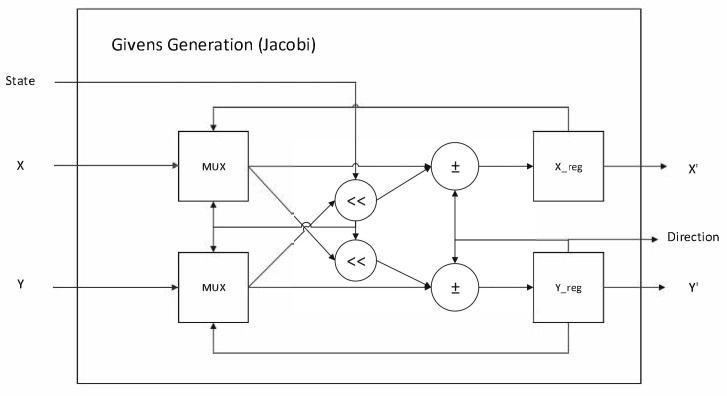
Givens Generation circuit architecture using the CORDIC algorithm.

The subsequent Jacobi module is the most complicated one in this pipelined data path. It is highly resource-intensive in hardware due to its core iterative rotations. Figure 12 shows the hardware design of the Jacobi module. The design kernel is a systolic array to iteratively drive the target off-diagonal elements toward zero via a sequence of trigonometric operations, i.e., Givens rotations. The GG (Givens Generation) unit determines the rotation direction—clockwise or counter-clockwise—based on the value of the element to be eliminated. Once the direction is determined, the signal is transmitted to other GR (Givens Rotation) units, enabling synchronized rotation for the remaining elements. By rotating the covariance matrix into a diagonal matrix, the desired eigenvalues are obtained, while the cumulative product of the rotation matrices yields the corresponding eigenvectors. To implement the GG and GR units, we employed a CORDIC (coordinate rotation digital computer)-based [33] approximation method to simplify the hardware design. Each CORDIC unit was configured as either the vector mode (for Givens Generation) or the rotation mode (for Givens Rotation). The former determines the rotation angle for nullification and passes the information to the downstream units for vector update. Figure 13 illustrates the design within a CORDIC module, which was configured into vector mode for Givens Generation. This architecture was implemented using only shifters, adders, and sign comparators. The design can be easily configured into rotation mode by the exclusion of the sign comparator. A design optimization measure is adopted here: instead of computing the rotation angle explicitly, the leading GG unit passes the rotation angle as a sequence of rotation directions, which can be either positive or negative. This approach dramatically simplifies both the control logic and the computational flow, enabling a high-performance hardware accelerator. This architectural optimization allows for the system to process a complete feature point every 88 clock cycles, enabling a peak processing latency of only 0.704 ms for 1000 points at 125 MHz in implementation.

### 4.7. Overall System Implementation

**Figure 14:**
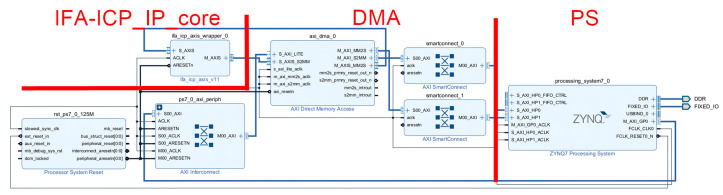
Hardware accelerator implementation in a PYNQ-Z2 FPGA platform.

Figure 14 illustrates the hardware implementation of the accelerator on the PYNQ-Z2 FPGA platform. The system architecture is partitioned into the Processing System (PS) and the Programmable Logic (PL). The PS consists of a dedicated dual-core ARM Cortex-A9 hard processor, which manages high-level scheduling and data flow. In contrast, the PL section houses the custom-designed IFA_ICP_IP and the AXI Direct Memory Access (DMA) IP module [34]. The DMA serves as a high-speed data interface, facilitating transaction-based transfers between the PS DDR memory and the hardware accelerator. The DMA core, along with other supporting modules such as AXI SmartConnect [35] (for bus width conversion), were generated automatically by using the design suite Block Design [36] supported in Vivado [37].

**Figure 15:**
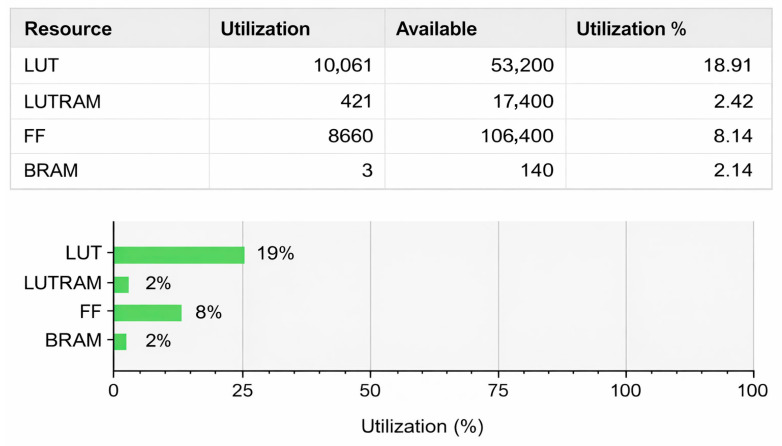
**FPGA** resource utilization ratio summary for hardware accelerator.

Figure 15 summarizes the resource utilization ratios in the FPGA device. The implementation accounts for less than 19% of the available LUT units, while the usage of memory units like FF and block RAM is even lower. This highlights that our proposed hardware accelerator design is both computationally efficient and lightweight in hardware resources. In addition, the data volume of point clouds is not particularly high, and the current DDR3 memory interface equipped in PYNQ Z2 suffices for the bandwidth demand. Therefore, porting to a more powerful SoC FPGA platform does not significantly help the throughput performance.

To further benchmark the hardware efficiency, we compared our design with a state-of-the-art FPGA-based LiDAR odometry accelerator, specifically the NLO-CSM scheme proposed by Hu et al. [21], which was also implemented on the same Zynq-7020 platform [38]. While NLO-CSM achieves a higher framerate of 111 FPS, it targets 2D indoor SLAM applications, processing only approximately 90 laser points per frame. In contrast, our IFA-ICP system addresses the far more computationally demanding 3D autonomous driving scenarios (KITTI dataset), handling over 15,000 points per frame. In addition, the 10.04 FPS throughput of the proposed design is actually bounded by the software execution. Referring to Figure 9, within the allotted 100ms timeframe, hardware execution accounts for only 20%. In other words, if we do not consider the coordination between hardware and software executions, hardware accelerator alone can deliver up to 5 times more throughput, i.e., 50FPS. Furthermore, despite handling complex 3D feature extraction and covariance matrix calculations, our hardware resource utilization (~19% LUTs) remains comparable to their 2D implementation (18.9% LUTs). This indicates that the proposed front-end acceleration strategy achieves high area efficiency while solving a significantly more complex 3D odometry problem.

**Figure 16:**
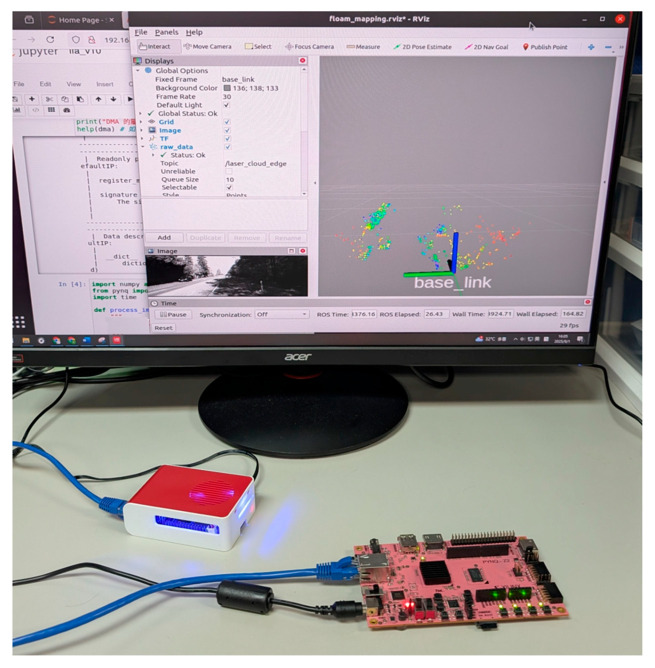
Entire SLAM system implementation based on HW/SW codesign.

Figure 16 shows a photo of an entire SLAM system implementation on a Raspberry Pi 4 + PYNQ-Z2 FPGA platform. The accelerator circuits operate at 125 MHz and demonstrate resilient accuracy, even with fixed-point arithmetic. Most critically, we achieved a final throughput of 10.04 FPS, a 54% performance uplift compared to the 6.54 FPS of the pure software implementation. This fulfills our primary research objective of meeting the stringent real-time processing demands (≥10 FPS) of practical applications.

### 4.8. Power Consumption Analysis and Discussion on Scalability and Portability

**Figure 17:**
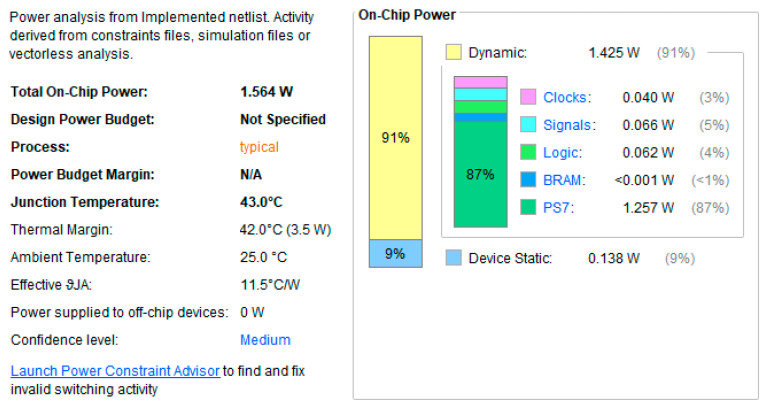
**FPGA** power consumption estimation report, and breakdown for the IFA-ICP hardware system on the PYNQ-Z2 platform.

To evaluate the energy efficiency of the proposed hardware accelerator, we performed a comprehensive power analysis using the Vivado Power Analysis tool [36]. The estimation is based on the target XC7Z020 FPGA operating at a clock frequency of 125 MHz. Under standard operating conditions (25 °C ambient temperature), the total on-chip power consumption was estimated at 1.637 W. As shown in the power breakdown Figure 17, the Processing System (PS) accounts for the majority of the power (1.542 W, 94%), which is consistent with the resource-intensive nature of the ROS middleware and software tasks running on the dual-core ARM processor. The custom hardware accelerator (PL section) exhibited extremely low dynamic power consumption (approximately 0.027 W), underscoring the efficiency of our lightweight architecture design. The junction temperature remained stable at 28.5 °C, providing a significant thermal margin of 56.5 °C before reaching the device limit.

## 5. Conclusions

In conclusion, in this paper, we investigated the image feature-assisted–Iterative Closest Point (ICP) scheme for odometry estimation and developed a novel scheme that leverages the richness of image information to compensate the sparsity issue of laser point cloud and thus enhance its matching accuracy. In addition, this scheme effectively reduces the computing complexity by point cloud screening. Various DSP schemes were also employed to improve the accuracy of odometry estimation. These optimization measures were implemented on an existing SLAM system, and the KITTI dataset for autonomous vehicles with ground truth was adopted to benchmark the performance. For the most critical translation error index, the simulation results indicate a 16.6% reduction, while the computing complexity also reduced by 63%. Based on this developed odometry estimation scheme, we further worked on the optimization of system implementation by using an HW/SW co-design strategy. A low-cost Raspberry Pi module served as the system host and was in charge of the most non-timing critical functions of the SLAM system. It was accompanied by a cost-effective PYNQ Z2 FPGA board as the hardware accelerator to implement time-critical tasks and expedite the estimation process. The implementation results indicate that our HW/SW co-design decisively elevated the performance of our SLAM algorithm. The processing throughput increased from 6.54 FPS to 10.04 FPS—a 54% enhancement. It fulfills the critical 10 FPS real-time requirement set by industry-standard LiDARs and benchmarks like the KITTI dataset.

## Figures and Tables

**Table 1:** Computer Spec. [22] of algorithm simulation verification.

CPU	OS	RAM	Storage
Intel^®^ Core i7-11370H @ 3.30 GHz	Ubuntu 20.04.4 LTS + ROS Noetic	32 GB DDR4 3200 MHz	500 GB M.2 NVMe™ PCIe^®^ 3.0 SSD

**Table 2:** Accuracy comparison in KITTI Evaluation sequence 01.

Algorithm	Translation Error (%)
A-LOAM	2.3812
FLOAM	2.5146
KISS-ICP	2.2921
Ours	1.8520

**Table 3:** Detailed accuracy comparison for different KITTI [10] dataset sequences.

	FLOAM		Ours	
Sequence	Translation Error (%)	Rotation Error (deg/m)	Translation Error (%)	Rotation Error (deg/m)
0	1.626	0.0074	1.0494	0.0058
1	2.5146	0.0049	1.8520	0.0048
2	1.6023	0.0051	1.1762	0.0050
3	1.1843	0.0060	1.1427	0.0049
4	1.0961	0.0034	1.0333	0.0043
5	0.6442	0.0032	0.6757	0.0041
6	0.6264	0.0034	0.6314	0.0033
7	0.7723	0.0058	0.7202	0.0079
8	1.1811	0.0046	1.1470	0.0041
**9**	1.0750	0.0040	0.9285	0.0037
**10**	1.3591	0.0046	1.0541	0.0039
**average**	1.2440	0.00477	1.0373	0.00471

**Table 4:** Overall execution time of algorithm in KITTI evaluation sequence 01.

Algorithm	Execution Time
A-LOAM	10.25×
FLOAM	3.59×
KISS-ICP	1.90×
Ours	1×

**Table 5:** Average numbers of points for Ceres Solvers between FLOAM and IFA-ICP.

Sequence	Average Numbers of Point for Ceres Solvers (Edge)	Reduction (%)	Average Numbers of Point for Ceres Solvers (Surface)	Reduction (%)
00	1862 → 412	77.87	1254 → 351	72.01
01	1221 → 253	79.28	956 → 201	78.97
02	1636 → 354	78.36	1025 → 264	74.24
06	1351 → 265	80.38	1325 → 428	67.70
Average	1518 → 321	78.85	1140 → 311	72.72

**Table 6:** HW/SW co-design system specifications.

	Raspberry Pi 4 Model B 8 GB [28]	PYNQ-Z2 [29]
SOC	Broadcom BCM2711	Zynq-7000 SoC XC7Z020-1CLG400C
CPU and FPGA	Quad-core Cortex-A72 (ARM v8) 64-bit SoC @ 1.5 GHz	Dual-core Cortex-A9 Processor @ 650 MHz Artix-7 (13,300 logic slices with four 6-input LUTs and 8 flip-flops, 630 KB BRAM, 220 DSP Slices)
Memory	8 GB LPDDR4	512 MB DDR3
Storage	128 GB micro SD (U3)	16 GB micro SD (U1)

**Table 7:** Execution time of each module on Raspberry Pi(ARM).

Module	Time (ms)	Percentage	HW Acceleration?
Gaussian blur	4.5888	3.0%	N
Sobel Filter	6.2514	4.1%	N
Adaptive Threshold	22.0117	**14.4** **%**	**Y**
Judgment	9.3152	6.1%	N
Built KD-Tree	25.3196	16.6%	N
ICP Pre-filter (Edge)	5.0944 (2.5472 × 2)	3.3%	N
PCA(Edge)	17.4558 (8.7279 × 2)	**11** **.4** **%**	**Y**
ICP Pre-filter (Surface)	7.9664 (3.9832 × 2)	5.2%	N
PCA(Surface)	25.7322 (12.8661 × 2)	**16.8** **%**	**Y**
Ceres Solver	14.5980 (7.2990 × 2)	9.6%	N
Other Process	14.4462	9.5%	N
Total	152.7797	100.0%	

## Data Availability

This research uses the public available KITTI vision benchmark suite [10] as the test bench.
